# Mechanistic Study of Antimicrobial Effectiveness of Cyclic Amphipathic Peptide [R_4_W_4_] against Methicillin-Resistant *Staphylococcus aureus* Clinical Isolates

**DOI:** 10.3390/antibiotics13060555

**Published:** 2024-06-13

**Authors:** Ajayi David Akinwale, Keykavous Parang, Rakesh Kumar Tiwari, Jason Yamaki

**Affiliations:** 1 Center for Targeted Drug Delivery, Department of Biomedical and Pharmaceutical Sciences, Harry and Diane Rinker Health Science Campus, Chapman University School of Pharmacy, Irvine, CA 92618, USAparang@chapman.edu (K.P.); 2 Department of Pharmacy Practice, Harry and Diane Rinker Health Science Campus, Chapman University School of Pharmacy, Irvine, CA 92618, USA; 3 Department of Basic Medical Sciences, College of Osteopathic Medicine of the Pacific–Northwest, Western University of Health Sciences, Lebanon, OR 97355, USA

**Keywords:** amphipathic, antimicrobial, cyclic peptide, mechanism of action, minimum bactericidal concentration (MBC), minimum inhibitory concentration (MIC)

## Abstract

Antimicrobial peptides (AMPs) are being explored as a potential strategy to combat antibiotic resistance due to their ability to reduce susceptibility to antibiotics. This study explored whether the [R_4_W_4_] peptide mode of action is bacteriostatic or bactericidal using modified two-fold serial dilution and evaluating the synergism between gentamicin and [R_4_W_4_] against *Escherichia coli* (*E. coli*) and methicillin-resistant *Staphylococcus aureus* (MRSA) by a checkered board assay. [R_4_W_4_] exhibited bactericidal activity against bacterial isolates (MBC/MIC ≤ 4), with a synergistic effect with gentamicin against *E. coli* (FICI = 0.3) but not against MRSA (FICI = 0.75). Moreover, we investigated the mechanism of action of [R_4_W_4_] against MRSA by applying biophysical assays to evaluate zeta potential, cytoplasmic membrane depolarization, and lipoteichoic acid (LTA) binding affinity. [R_4_W_4_] at a 16 mg/mL concentration stabilized the zeta potential of MRSA −31 ± 0.88 mV to −8.37 mV. Also, [R_4_W_4_] at 2 × MIC and 16 × MIC revealed a membrane perturbation process associated with concentration-dependent effects. Lastly, in the presence of BODIPY-TR-cadaverine (BC) fluorescence dyes, [R_4_W_4_] exhibited binding affinity to LTA comparable with melittin, the positive control. In addition, the antibacterial activity of [R_4_W_4_] against MRSA remained unchanged in the absence and presence of LTA, with an MIC of 8 µg/mL. Therefore, the [R_4_W_4_] mechanism of action is deemed bactericidal, involving interaction with bacterial cell membranes, causing concentration-dependent membrane perturbation. Additionally, after 30 serial passages, there was a modest increment of MRSA strains resistant to [R_4_W_4_] and a change in antibacterial effectiveness MIC [R_4_W_4_] and vancomycin by 8 and 4 folds with a slight change in Levofloxacin MIC 1 to 2 µg/mL. These data suggest that [R_4_W_4_] warrants further consideration as a potential AMP.

## 1. Introduction

Multidrug-resistant bacteria pose a significant threat to public health, exerting significant economic and clinical impacts, including high cost of treatment, significant morbidity and mortality, and increased length of hospitalization in patients infected with these organisms [1,2,3,4]. The potential source of multidrug-resistant bacteria may stem from hospital-acquired infections (HAIs), also known as nosocomial infections, which can lead to increased community transmission [5,6,7]. Additionally, infectious diseases rank as the second leading killer globally and fourth in the United States [8]. In the United States, nearly two million people are infected with multidrug-resistant bacterial pathogens, resulting in over 20,000 deaths annually [9]. Moreover, both the World Health Organization (WHO) and Centers for Disease Control and Prevention (CDC) forecast the rise in multidrug-resistant bacterial pathogens referred to as “superbugs” [10,11,12,13]. Examples include *Enterococcus* spp., *Staphylococcus aureus*, *Klebsiella pneumoniae*, *Acinetobacter* spp., *Pseudomonas aeruginosa*, and *Enterobacter* spp., which are common bacteria responsible for reported antimicrobial-resistant infections [14,15].

One promising strategy to combat antibiotic-resistant organisms is the use of antimicrobial peptides (AMPs). The efficacy of AMPs is attributed, in part, to their decreased susceptibility to bacteria resistance, as their innate evolutionary structures differ significantly from conventional antibiotics [16,17]. AMPs can function as fast-acting broad-spectrum antimicrobial agents, having been demonstrated to have activity against bacteria, fungi, viruses, and certain cancers [18]. Generally, AMPs are polypeptide sequences comprised of cationic amino acids, predominantly arginine, lysine, and hydrophobic amino acids with direct antimicrobial activity [19]. Many AMPs are recognized as host defense peptides, a vital component of the innate immune system and first-line defense against foreign invaders in plants, invertebrates, and vertebrates [20,21]. 

One proposed model for the mechanisms of action (MOA) of AMPs involves their potential membrane-destabilizing and membrane-permeabilizing abilities due to their interfacial activity as a critical function [22]. In this model, the AMPs’ primary function depends on the interfacial activity rather than the specific amino acid components or three-dimensional structures [23]. The interfacial activity allows the peptides to partition into membrane–water interfaces, likely altering lipid structures, enhancing ion leakage, and causing a loss of biological membrane structure and functions [24]. Moreover, AMPs have been demonstrated to exhibit antimicrobial effects with specificity against bacterial pathogens [25]. The membranes of eukaryotic cells are made up of uncharged phospholipids, phosphatidylethanolamine, phosphatidylcholine, sphingomyelins, and cholesterol [26]. In contrast, the membranes of many bacteria are composed of negatively charged lipid groups such as phosphatidylglycerol, lipopolysaccharides, and cardiolipins [27]. Thus, the capability of positively charged AMPs interacting with anions on the surface of microbial membranes confers a crucial selective action in destroying microbes [27].

Clinically, vancomycin and colistin are examples of AMPs that are commercially available for the treatment of multidrug-resistant bacteria, with vancomycin having activity against Gram-positive and colistin having activity against Gram-negative bacteria [28]. One promising synthetic cationic amphiphilic cyclic AMP is [R_4_W_4_], which consists of four arginine (R) and four tryptophan (W) amino acids, exhibiting antibacterial activity against multidrug-resistant pathogens [19,29]. While the precise mechanisms of action of the [R_4_W_4_] peptide as an antibacterial agent have not been fully elucidated, prior research has demonstrated antibacterial activity against Gram-positive and Gram-negative bacterial pathogens [30,31]. Nonetheless, investigations into the antibacterial activity of [R_4_W_4_] against clinical strains of multidrug-resistant bacteria and mechanistic studies of its mode of action have yet to be explored [27].

In this study, we aimed to evaluate [R_4_W_4_] against various multidrug-resistant clinical isolates of MRSA and selected pathogens, its stability and antibacterial activity under various conditions, the ability for resistance generation by organisms, and potential mechanisms of antibacterial activity. We evaluated the antibacterial efficacy of [R_4_W_4_] against twelve clinical drug-resistant bacteria isolates distinct from the previously reported strains [28,29,30]. As a prospective drug candidate, we explored the stability of [R_4_W_4_] to heat exposure, human serum and plasma enzymes, and sensitivity to physiological salts. Furthermore, we examined the synergistic effects of [R_4_W_4_] and gentamicin against MRSA and *E. coli*. Additionally, we applied serial passage assay to show if [R_4_W_4_]-resistant MRSA strain will display cross-resistance to a variety of clinically approved antibiotics with various modes of action [32]. Moreover, the study delved into mechanistic investigations of the antibacterial activity of [R_4_W_4_]. A comprehensive understanding of the mechanisms of the antibacterial action of [R_4_W_4_] will provide a foundation for the discovery of more potent AMPs.

## 2. Results

### 2.1. Antibacterial Effectiveness of [R_4_W_4_]

#### 2.1.1. Bacteriostatic vs. Bactericidal Evaluation

The antibacterial activity of [R_4_W_4_] was evaluated against nine clinical isolates of MRSA, one laboratory strains of *Bacillus subtilis* (ATCC 21332), a *Clostridioides difficile* (ATCC 9689), and a vancomycin-resistant *Enterococcus faecium* (VRE) strain by microbroth dilution assay using vancomycin as a control, as shown in Table 1. [R_4_W_4_] exhibited broad-spectrum activity against both Gram-positive and Gram-negative bacteria, demonstrating minimum inhibitory concentrations (MIC) values ranging from 2 to 32 µg/mL. While vancomycin was not active against *Enterococcus faecium* (VRE), [R_4_W_4_] exhibited a MIC value of 16 µg/mL. The vancomycin MICs for *Clostridioides difficile* and the clinical MRSA strains demonstrate susceptibility based on established MIC breakpoints. However, there are no MIC interpretations or breakpoints available for [R_4_W_4_], as it is an investigational agent. The corresponding MIC and minimum bactericidal concentrations (MBC) for each tested bacteria isolate are shown in Table 1. The antibacterial activity of [R_4_W_4_] and vancomycin varied depending on the bacterial isolates. For example, [R_4_W_4_] exhibited MIC values ranging from 2 to 32 μg/mL against all MRSA strains, while it showed a MIC of 16 μg/mL against the VRE strain. [R_4_W_4_] showed more potent against MRSA clinical isolates from the blood than lungs with MIC of 2–4 and 16–32 µg/mL, respectively, whereas MRSA isolates from the wound showed a wide range of MIC of 8–64 µg/mL. [R_4_W_4_] was efficacious against *B. subtillis* and *C. difficile* with MIC of 4 and 2 µg/mL, respectively. The MBCs of [R_4_W_4_] and vancomycin against all the strains tested showed a 2–4-fold increase in their MIC values. According to Pankey’s definition, the antibacterial effectiveness of [R_4_W_4_] as a bacteriostatic or bactericidal agent was calculated using the ratio of MBC/MIC [33]. The MBC/MIC ratios range against all tested bacterial isolates were 2 to 4 fold for [R_4_W_4_] and 1–2 fold for vancomycin. Hence, the activities of [R_4_W_4_] and vancomycin antibacterial agents were bactericidal.

#### 2.1.2. Evaluation of Time-of-Kill

**Figure 1:**
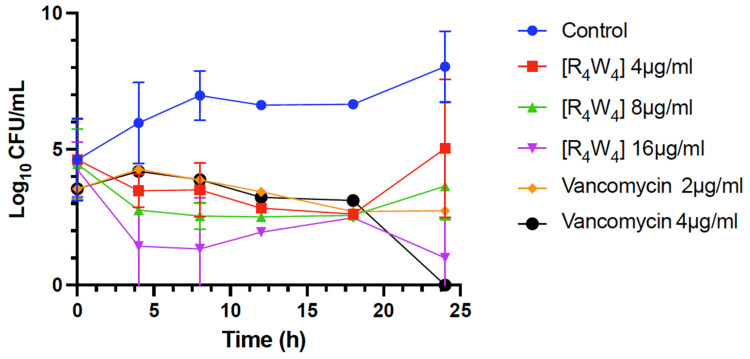
Time of kill for [R_4_W_4_] and vancomycin against MRSA (LAC). Note: data points are based on triplicate independent experiments, except for time points 12 and 18 h, which were only included once.

Time–kill assays were performed with MRSA using [R_4_W_4_] and vancomycin at concentrations of 4 µg/mL and 1 µg/mL, respectively, which corresponded to 1 × MIC. The data revealed the reduction in viable bacterial MRSA in Mueller–Hinton Broth (MHB) after adding 1 × MIC, 2 × MIC, and 4 × MIC of [R_4_W_4_] (99.67–99.87%) and vancomycin (90.50–99.58%). Control incubations were carried out without peptide [R_4_W_4_] and vancomycin. The results demonstrate rapid and highly effective antimicrobial activity, with a percent reduction of >99% for [R_4_W_4_] and >90% for vancomycin at MIC (Table 2). The time–kill curve is depicted in Figure 1. Notably, at 4 × MIC with [R_4_W_4_], a significantly fast reduction in colony forming unit (CFU) was observed after 4 h of incubation compared to the control, as well as to vancomycin concentrations and [R_4_W_4_] at 1 × and 2 × MICs. However, by 12 h, there was a slight increase in growth, with bacteria remaining viable up to 26 h. On the other hand, no viable bacteria were recovered at 26 h with vancomycin at 4 × MIC with a 99.58 percent reduction (Figure 1).

#### 2.1.3. Synergism between [R_4_W_4_] and Gentamicin

**Figure 2:**
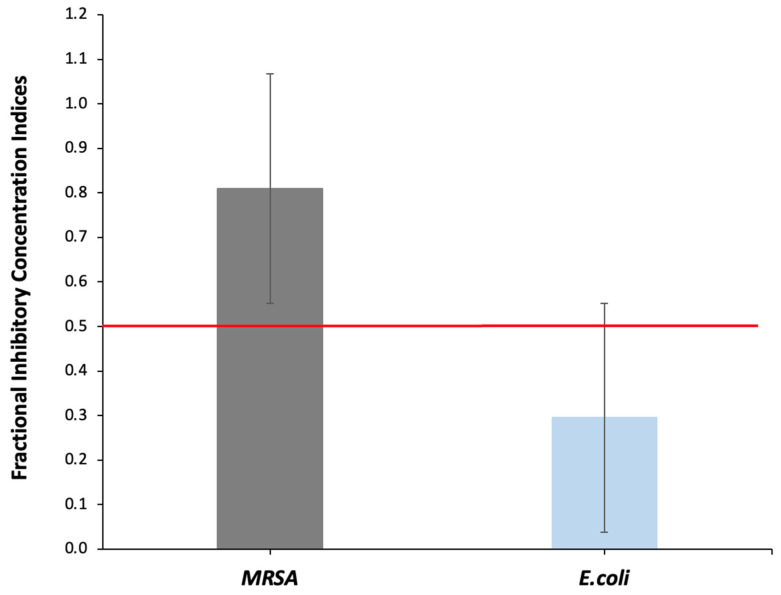
Mean fractional inhibitory concentration indices (FICI) of MRSA and *E. coli* with the combination of [R_4_W_4_] and gentamicin at 0.75 and 0.38 µg/mL, respectively. Results are from two independent experiments performed in triplicate. The FICI synergy definition is depicted by the red line.

The fractional inhibitory concentration index (FICI) of [R_4_W_4_] and gentamicin was evaluated using a checkered board assay to determine whether a synergistic effect exists between the two antibacterial agents against MRSA and *E. coli*. Table 3 shows no synergistic effect between [R_4_W_4_] and gentamicin against MRSA (FICI = 0.81), but rather an additive effect of two antibiotics, resulting in a reduction in MIC values of [R_4_W_4_] by 4 fold and approximately ~2 fold for gentamicin in combination. Conversely, [R_4_W_4_] and gentamicin exhibited a synergistic effect on *E. coli* (FICI = 0.30), resulting in a reduction in MIC value of [R_4_W_4_] by 2 fold and approximately 4 fold for gentamicin in combination. Figure 2 depicts the mean FICI from multiple experiments and where the FICI for MRSA and *E. coli* fall in relation to the FICI synergy definition (red line).

#### 2.1.4. Bacterial Resistant Development to [R_4_W_4_] 

**Figure 3:**
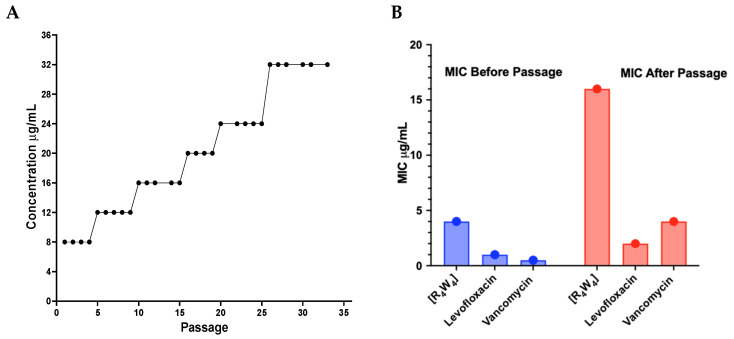
(**A**) Modest increment of [R_4_W_4_] and the MRSA strain capability to develop resistance to [R_4_W_4_] high concentration. At the end of the experiment, the antibacterial effectiveness of [R_4_W_4_] and other clinically approved antibacterial agents was determined against the MRSA-acquired-resistant strain. (**B**) The change in the MIC of antibacterial agents [R_4_W_4_], levofloxacin, and vancomycin increased to 16, 2, and 4 folds, respectively. The MIC experiment was performed in duplicate, and the MICs obtained were the same for each experiment.

The in vitro resistance development of MRSA USA300 to [R_4_W_4_] was determined by serial passage assay [34]. The capability of MRSA bacteria to acquire resistance in the presence of a stepwise increasing concentration of [R4W4] was performed by daily serial passaging of the bacteria for 35 passages (Figure 3A). At the end of the serial passage assay, the growth of MRSA USA300 was achieved in the final concentration of 32 µg/mL [R_4_W_4_]. Subsequently, a standard MIC assay was conducted using the selected resistant serial passaged bacteria, comparing [R_4_W_4_], vancomycin, and levofloxacin MICs to those of the index strain that did not undergo serial passage (Figure 3B). We found that the [R_4_W_4_] MIC in these subsequent experiments was 16 µg/mL compared to the 4 µg/mL of the index isolate and that levofloxacin MICs minimally changed from 1 µg/mL to 2 µg/mL. However, the MICs increased in the serial passaged isolate to 4 µg/mL for vancomycin from 0.5 µg/mL. The significant increase in vancomycin MICs suggests the potential for cross-resistance development with continuous exposure to [R_4_W_4_]. The minimal change in levofloxacin MICs suggests a more specific resistance mechanism related to vancomycin and [R_4_W_4_].

#### 2.1.5. Evaluation of [R_4_W_4_] Stability Assays

##### Human Serum Stability and Plasma Stability

**Figure 4:**
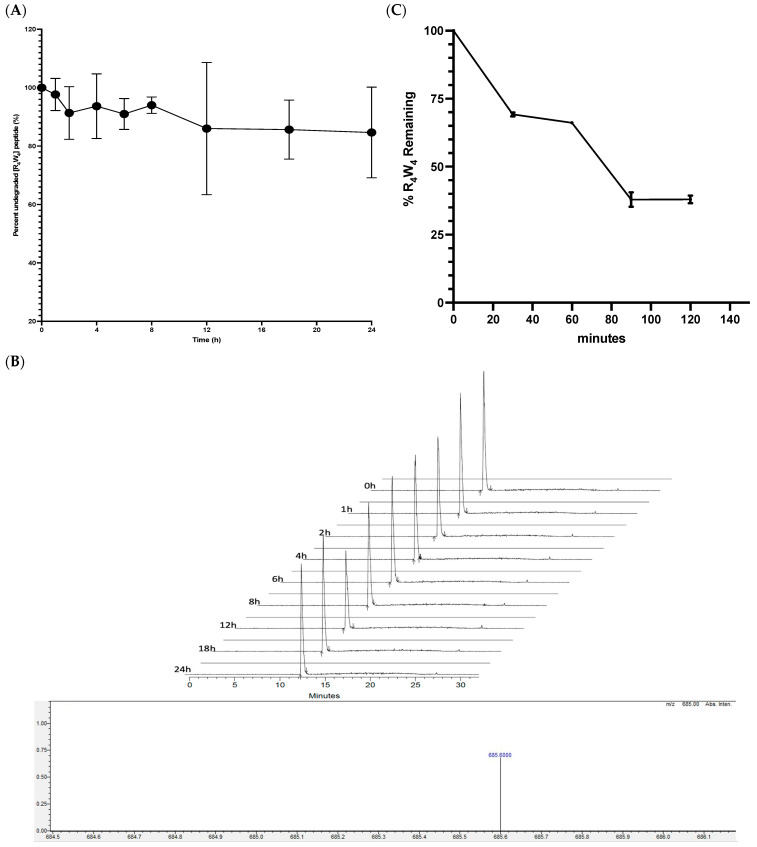
(**A**) In vitro serum stability profile of [R_4_W_4_] in 25% fresh human serum is depicted. The percentage of intact peptide remaining over a period of 20 h was measured by LC-MS at peak mz 685 (corresponding to M^2+^) and retention time of 12.5 min. The data obtained are from three independent experiments. (**B**) Representative image of [R_4_W_4_] 25% serum stability spectra and compound mass. (**C**) In vitro stability profile of [R_4_W_4_] in 100% plasma, the percentage of the intact compound at each timepoint is depicted over 120 min.

The proteolytic susceptibility of [R_4_W_4_] in human serum was determined by monitoring the percentage of remaining [R_4_W_4_] after incubation with 25% human serum at 37 °C, using LC-MS (Figure 4). Based on the linearity curve of [R_4_W_4_], the limit of detection (LOD) and limit of quantification (LOQ) were determined to be 6.4 and 19 μg/mL, respectively. After 1 h of incubation, approximately 99% of the peptide was found intact in human serum. Furthermore, after 2, 6, 12, and 20 h, the remaining percentages of [R4W4] were 93, 80, 77, and 76%, respectively. It is evident that after 24 h, we did not see any further decrease in [R_4_W_4_], presumably due to the lower serum protein concentration in 25% serum. Seeing that a significant amount remains at 20 h, it will be difficult to calculate its half-life time. 

Conversely, when exploring the stability in 100% human plasma, which is more closely a surrogate for blood stability compared to 25% serum, we found that stability was significantly shorter, with a half-life of approximately 86 min. This could be due to higher protein and enzyme concentrations in 100% plasma compared to 25% (Figure 4C).

##### Physiological Salts and Thermal Stability Tests

**Figure 5:**
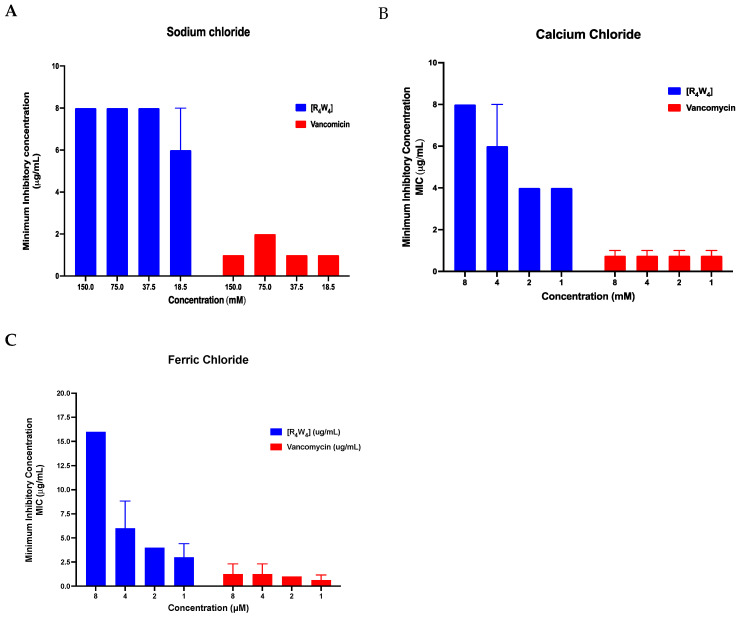
Effect of various physiological salt concentrations on [R_4_W_4_] such as (**A**) sodium chloride, (**B**) calcium chloride, and (**C**) ferric chloride. Note: bars with no error bars are the result of duplicate experiments resulting in the same values.

The ability of [R_4_W_4_] to continue to exert antibacterial activity against MRSA was investigated under different physiological salts at various concentrations, as reflected by determining the MIC. Figure 4 illustrates the MIC of [R_4_W_4_] and vancomycin against the MRSA LAC strain when subjected to different physiological salt concentrations of sodium chloride (18.5, 75, 37.5, and 150 mM), calcium chloride (1, 2, and 4 mM), and ferric chloride (1, 2, 4, and 8 μM). Under normal physiological conditions, the MIC for [R4W4] and vancomycin are 4 µg/mL and 1 µg/mL, respectively. However, in the presence of higher concentrations of saline, the MIC of cyclic antibacterial [R_4_W_4_] and vancomycin slightly change, ranging from 6 to 8 µg/mL and 1 to 2 µg/mL, respectively (Figure 5A). Notably, under the influence of calcium chloride salt, the MIC of vancomycin remains the same (0.75 µg/mL), while the MIC of [R_4_W_4_] varies between 4 and 8 µg/mL (Figure 5B). Similarly, under the influence of ferric chloride salt, the MIC of [R_4_W_4_] increases proportionally with the concentration of ferric chloride, while the vancomycin MIC ranges between 0.625 and 1.25 µg/mL (Figure 5C).

**Figure 6:**
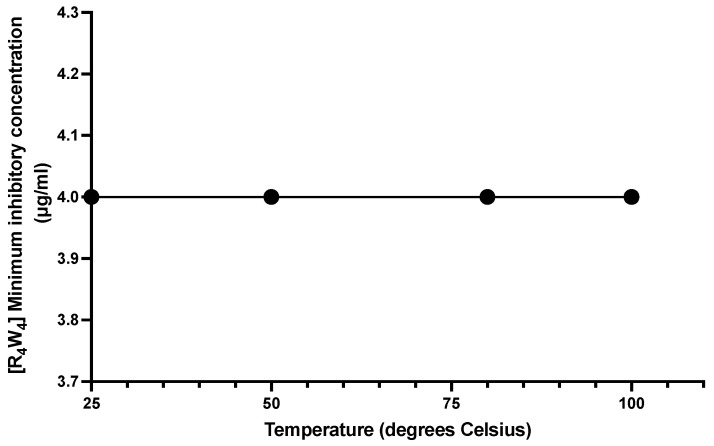
[R_4_W_4_] thermal stability showing unchanged MIC after exposure to temperatures 25, 50, 75, and 100 °C.

The thermal stability of the peptide was conducted by subjecting [R_4_W_4_] to various temperatures and then evaluating its MIC against MRSA LAC strain. Figure 6 depicts no change in MIC after thermal exposure of [R_4_W_4_] at 25, 50, 75, and 100 °C, with a consistent MIC of 4 μg/mL. Demonstrating that, despite increasing high temperatures, [R_4_W_4_] retained its antibacterial activity, which suggests a promising property of temperature stability.

#### 2.1.6. Surface Charge Neutralization of MRSA LAC USA300

**Figure 7:**
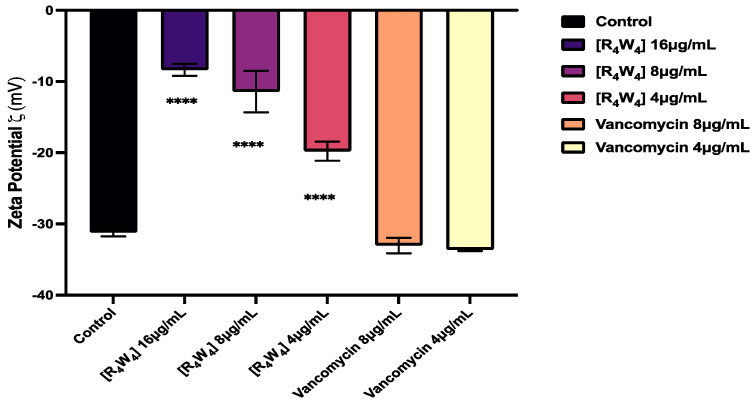
Effect of [R_4_W_4_] and vancomycin on zeta potential properties of Gram-positive MRSA after being treated with antibacterial agents at concentrations (4, 8, and 16 mg/mL). The chart shows a neutral surface net charge, and each value represents the mean of triplicate determinations with a *p*-value (**** *p* < 0.0001).

We assessed the zeta potential to monitor the effect of [R_4_W_4_] on the membrane surface charge of MRSA, with vancomycin as a control. In the absence of [R_4_W_4_], the MRSA LAC strain exhibited an electromobility potential of −31 ± 0.88 mV. Upon the addition of increasing concentrations of [R_4_W_4_] up to 16 μg/mL (4 × MIC), the zeta potential values rose and stabilized at −8.37 mV. The concentration of [R_4_W_4_] required to achieve the neutralization negative surface charge was 8 μg/mL (2 × MIC). As expected, the zeta potential at 16 µg/mL vancomycin concentration increased to −33.03 mV, suggesting no significant change compared to the untreated MRSA LAC strain (Figure 7). Comparisons of zeta potential comparisons between groups show statistically significant differences at concentrations relative to control, suggesting that [R_4_W_4_] probably neutralizes negative surface charge, thereby perturbing the bacteria cell membrane. This mechanism is typical for many antimicrobial peptides and certain antibiotics, which target the bacterial membrane by interacting with lipoteichoic acids in Gram-positive bacteria. By neutralizing the negative charge, these compounds disrupt the electrostatic balance and integrity of the cell membrane, leading to increased permeability, leakage, and, ultimately, bacterial death.

#### 2.1.7. Membrane Depolarization Potential

**Figure 8:**
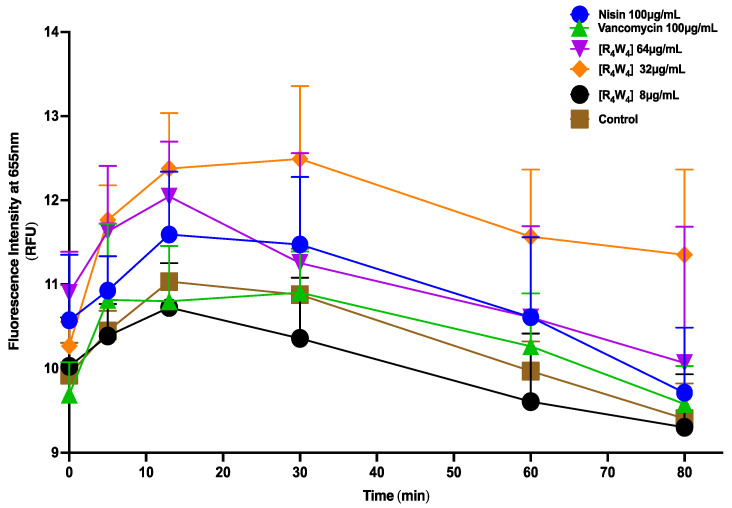
The membrane depolarization of MRSA USA300 with Disc_3_(5) with [R_4_W_4_] at 8, 32, and 64 μg/mL and 100 µg/mL nisin and vancomycin.

The membrane depolarization potential of [R_4_W_4_] at 2 × MIC and 16 × MIC was determined against MRSA using the membrane potential sensitive fluorescence dye 3,3”-dipropylthiadicarbocyanine iodine (DisC_3_(5)), with nisin at 100 μg/mL and vancomycin at 100 μg/mL, serving as positive and negative controls, respectively, against MRSA USA300. The DisC_3_(5) cationic probe accumulates in the bacteria cytoplasmic membrane and, upon membrane disruption, is released into the buffer, which can be detected by fluorescence intensity. Figure 8 shows that the treatment of MRSA with [R_4_W_4_] at 16 × MIC in the presence of the fluorescence dye DisC_3_(5) resulted in an enhanced fluorescence signal that surpassed that of nisin. In contrast, at the treatment of [R_4_W_4_] 2 × MIC, the fluorescence decreases below that of vancomycin and control, possibly due to concentration-dependent membrane depolarization potential effects. A significant difference (*p* = 0.0007) was observed between the control and [R_4_W_4_] at 32 and 64 µg/mL and nisin at 100 µg/mL. As expected, there was no significant difference between control and vancomycin 100 µg/mL.

#### 2.1.8. Lipoteichoic Acid (LTA) Binding Assay to MRSA

Two methods were utilized to evaluate the interaction of [R_4_W_4_] with LTA. The first method is the BODIPY-TR-cadaverine (BC) displacement method, using BODIPY-TR-cadaverine fluorescent dye, while the second method evaluates the MIC of MRSA in the presence and absence of LTA.

##### BC Displacement Method

**Figure 9:**
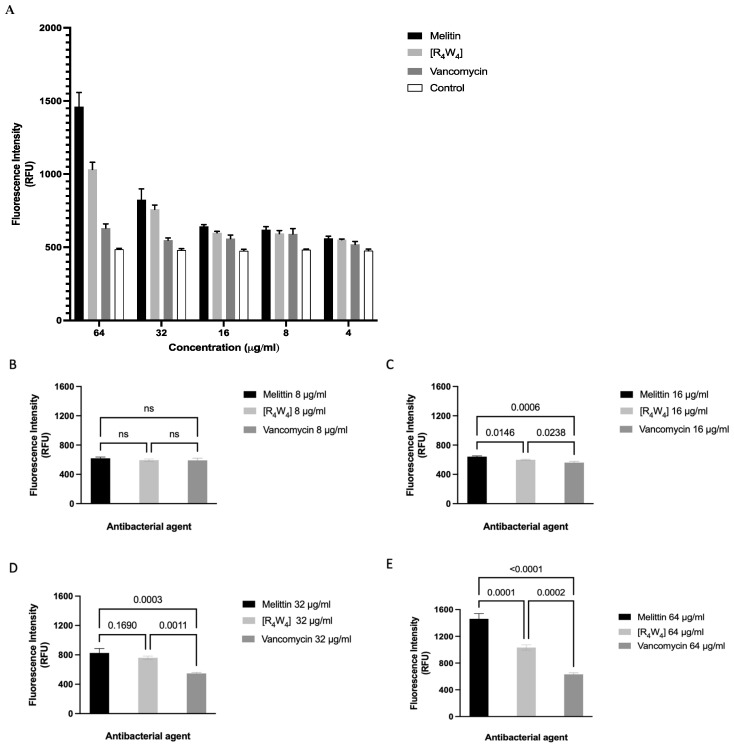
(**A**) LTA binding affinity of [R_4_W_4_], melittin, and vancomycin using BODIPY-TR-cadaverine fluorescent dye. Melittin and vancomycin are positive and negative controls, respectively. (**B**) There is no statistically significant difference between vancomycin [R_4_W_4_] and melittin at 8 μg/mL antibacterial concentrations. (**C**,**D**) There is a statistically significant difference when the negative control is compared with [R_4_W_4_] and melittin at 16 μg/mL and 32 μg/mL. (**E**) At 64 μg/mL there was a significant difference between melittin compared to [R_4_W_4_] and vancomycin, both of which resulted in no additional fluorescence compared to 32 μg/mL.

The binding affinities of [R_4_W_4_], melittin, and vancomycin to LTA were evaluated using BC fluorescent dye. Figure 9 indicates that melittin (the positive control) showed the highest binding affinity to LTA at 64, 32, 16, and 8 μg/mL, followed by [R_4_W_4_]. In contrast, the binding affinity of vancomycin (negative control), as measured by fluorescence intensity, was similar to the buffer treated with BC fluorescence dye and LTA without the antibacterial agents (control), which is expected for vancomycin since it is not known to bind to LTA.

##### Bacterial Viable Growth in the Presence of LTA

**Figure 10:**
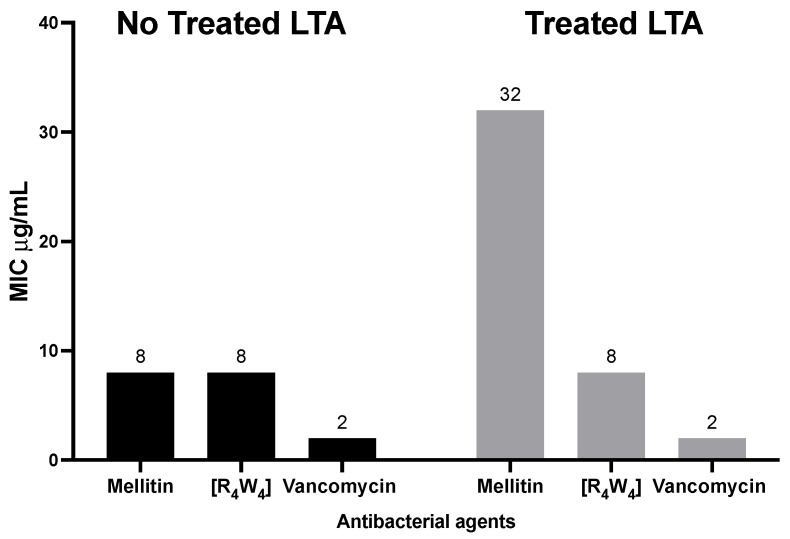
Influence of LTA binding affinity on the antibacterial effectiveness of melittin, [R_4_W_4_], and vancomycin in the presence of MRSA USA300. The experiment was carried out in duplicate, with MIC results the same each time.

The antibacterial activity of melittin, [R_4_W_4_], and vancomycin were determined in the presence and absence of LTA. Figure 10 shows that the MIC of melittin and [R_4_W_4_] in the presence of LTA are 32 μg/mL and 8 μg/mL, respectively, compared to 8 μg/mL MIC in the absence of LTA. As expected, the MIC of the negative control vancomycin remains unchanged at 2 μg/mL in both the presence and absence of LTA.

## 3. Discussion

The findings presented in this study demonstrate the potential of [R_4_W_4_], a synthetic cationic amphiphilic cyclic antimicrobial peptide, as a promising agent against multidrug-resistant bacteria. The evaluation of [R_4_W_4_] demonstrated effective antibacterial activity against various antibiotic-resistant bacterial clinical isolates, including isolates of MRSA and vancomycin-resistant *Enterococcus faecium* (VRE). Previously, [R_4_W_4_] had been reported to have activity against Gram-negative and positive laboratory strains of bacteria, which is why the study explored the potential mechanisms of action of [R_4_W_4_] [29]. In the present study, we determined the MIC and MBC of twelve clinically reported bacterial isolates, comprising nine MRSA strains, a vancomycin-resistant *Enterococcus faecium*, *Clostridioides difficile*, and a laboratory strain of *Bacillus subtilis*. We used the standard definitions of minimum inhibitory concentration (MIC), which is the lowest concentration that prevents visible growth of bacteria after 24 h, and the minimum bactericidal concentration (MBC) as the concentration that achieves a reduction in bacterial density by 1000 fold after 24 h. Based on the MIC and MBC results, we could categorize the activity of [R_4_W_4_] as bactericidal against the clinical strains, indicating potent antibacterial effectiveness. It is important to realize that whether an agent will be highly active or not does not depend on whether it is bactericidal or bacteriostatic. Rather, these definitions are based on the MIC and MBC ratios. By definition, a bactericidal agent will have a ratio of MBC-to-MIC ≤ 4 and bacteriostatic > 4. While a bactericidal agent will have an MBC closer to the MIC, and thus having a lower concentration required to achieve a 1000-fold reduction in bacterial density [35].

In addition, the kinetic time of kill of [R_4_W_4_] and vancomycin was performed to obtain the lethal rate and percent reduction and compare the results with the clinically approved glycopeptide vancomycin [33]. We found that vancomycin demonstrated a more potent antibacterial activity against the MRSA strains and *B. subtilis*. Moreover, the time-of-kill kinetic study of the [R_4_W_4_] showed a concentration-dependent reduction in viable bacteria by a percent reduction of >99% at 1 × MIC, 2 × MIC, and 4 × MIC against MRSA within 24 h, substantiating the notion that [R_4_W_4_] causes a complete eradication of viable bacteria within the time frame as a bactericidal agent.

Varying outcomes emerged when assessing the synergistic potential of [R_4_W_4_] and gentamicin against MRSA and *E. coli*. The fractional inhibitory concentration index (FICI) value ≤ 0.5 is considered synergistic [36,37]. The FICI values of [R_4_W_4_] and gentamicin against MRSA and *E. coli* were 0.81 and 0.30, respectively. Thus, the combination of [R_4_W_4_] and gentamicin produced a partial synergistic effect against MRSA and demonstrated clear synergistic antibacterial activity against *E. coli*, as substantiated by the lower FICI of 0.30. This demonstrates the combined action of [R_4_W_4_] and gentamicin could potentially be a valuable strategy for combating antibiotic-resistant Gram-negative bacteria [38]. This observation aligns with a previous study that demonstrated that the physical mixture of [R_4_W_4_] and levofloxacin had a synergistic effect against *Pseudomonas aeruginosa* [30]. Further investigation is required to explore the combination of [R_4_W_4_] and other approved clinical antibiotics against multidrug-resistant Gram-negative bacteria.

We demonstrated that under continuous pressure with serial passaging isolates in [R_4_W_4_], the MICs for [R_4_W_4_] increased approximately every 5–7 days and reached 32 μg/mL after 35 passages. With the increase in [R_4_W_4_] MICs, vancomycin MICs also increased, suggesting that with a serial passage, the MRSA USA300 strain under investigation has developed resistance to both [R_4_W_4_] and vancomycin likely due to their similar mechanism of action on the bacteria cell wall and membrane, with a decrease in antibacterial effectiveness MIC increasing 8 and 4 folds, respectively [39]. On the other hand, the levofloxacin MIC slightly changed two fold, likely due to its differing mechanism of action on DNA gyrase rather than cell wall and membrane [40]. Understanding these resistance patterns is critical for developing effective treatment strategies against microorganisms, especially in the context of rising antimicrobial resistance. The findings underscore the need for continuous monitoring of resistance trends and the potential impact of new antibiotics on existing treatment regimens. The ability of MRSA to adapt to [R4W4] over time emphasizes the ever-changing nature of bacterial resistance and the importance of innovative approaches to combat persistent infections.

The stability of [R_4_W_4_] human serum peptide degradation was determined with 25% human serum at 37 °C and demonstrated a half-life that exceeded 20 h. A previous study demonstrated that AMPs with terminal amine group have short half-lives due to carboxylate enzyme attack in human serum while capping with hexamer contributed to a slight change in the extended half-life, but in contrast, cyclized peptides had a significantly longer half-life [41]. Here, [R_4_W_4_] a cyclized peptide remained over 70% of the undegraded peptide after 20 h in 25% serum. When tested in 100% human plasma, we found a significantly shorter half-life of approximately 86 min. Many previous studies have used 25% human serum rather than 100% for testing the stability of cyclic peptides in which they found long half-lives, suggesting that perhaps the concentrations of enzymes in 25% may not be representative of actual in vivo concentrations and that use of 100% plasma may be a more in vivo representative approach [41,42,43].

Furthermore, the antibacterial activity of [R_4_W_4_] against MRSA was assessed using physiological salts at varying concentrations. The MIC of [R_4_W_4_] only slightly changed by the presence of sodium chloride salt (18.5–150 mM), indicating that its antibacterial activity is not significantly affected by physiological salts such as sodium chloride. However, there was a notable alteration in the antibacterial activity, as noted by changes in the MIC, when [R_4_W_4_] was subjected to high concentrations of calcium chloride (4 mM) and ferric chloride (8 mM). This finding aligns with a prior study indicating a decrease in antimicrobial activity of a short 6-peptide in a calcium salt medium [44]. Additionally, exposure of [R_4_W_4_] to extreme temperatures up to 100 °C did not alter its antibacterial activity, highlighting its stability and potential efficacy in maintaining therapeutic effects under physiologic temperatures and extreme storage conditions.

Moreover, the assessment of alterations in the negative surface charge of MRSA membranes was conducted by the change in zeta potential measurement, a valuable parameter for gauging the electrical potential at the particle’s slipping plane, which is defined as the distance where the structure with its chemically bound water and ions moves in bulk through a solution. In the absence of [R_4_W_4_], MRSA displayed a negative zeta potential of −31 ± 0.88 mV, with this inherent electronegative surface charge stabilizing at −8.37 mV following treatment with [R_4_W_4_] up to a concentration of 16 µg/mL. When compared with vancomycin, the concentration-dependent effect of [R_4_W_4_] on the MRSA membrane potential illustrates the specificity of [R_4_W_4_] in altering the electrostatic properties of MRSA [45,46]. The results of our study closely align with those reported by Halder et al. [47], indicating that [R_4_W_4_] similarly modulated the electrostatic properties and membrane potential in MRSA. Consistent with their findings with the surface-active compounds cetyl trimethyl ammonium bromide (CTAB) and polymyxin B, we observed a concentration-dependent increase in surface charge neutralization following [R_4_W_4_] treatment, as evidenced by the shift from an initial negative zeta potential to a less negative value. This consistency in zeta potential trends underscores the reproducibility of the MRSA plasma membrane being altered by membrane-acting agents such as AMPs, which we hypothesized [R_4_W_4_] acts upon [47].

We evaluated the impact of [R_4_W_4_] on MRSA bacterial membrane potential depolarization by fluorimetry utilizing Disc3(5) fluorescence dye. Under conditions where the membrane remains intact, the Disc3(5) probe accumulates within the bacterial cytoplasmic membrane and undergoes self-quenching; however, when membrane integrity is compromised, the fluorescence dye is released, resulting in increased fluorescence [48]. Following treatment with [R_4_W_4_] at 16 × MIC, the fluorescence increased, indicating a concentration-dependent membrane disruption effect. Fluorescence at 16 × MIC exceeded the positive control, whereas at 2 × MIC, membrane potential depolarization resembles that of the control. Halder et al. found that both surface-active compounds CTAB and polymyxin B resulted in the accumulation of crystal violet due to membrane disruption in *S aureus* [47]. Similarly, we found that [R_4_W_4_] led to increased fluorescence when treated with [R_4_W_4_] due to the release of DisC_3_(5) from the intracellular compartment, suggesting [R_4_W_4_] leads to cytoplasmic membrane disruption dependent on [R_4_W_4_] concentration.

Furthermore, the binding affinity of [R_4_W_4_] to LTA was evaluated by the BODIPY-TR-cadaverine (BC) fluorescent dye displacement method, and the assessment of changes in antibacterial activity (MIC) both in the presence and absence of LTA [49]. The data indicated a statistically significant difference when vancomycin, which does not bind to LTA, is compared with [R_4_W_4_] and melittin (positive control) at antibacterial concentrations above 16 µg/mL. However, in the treatment of MRSA both in the presence and absence of LTA (100 µg/mL), the binding affinity predicted for [R_4_W_4_] did not translate into a change in the antibacterial effectiveness (MIC). For example, the data show that while the antibacterial activity of melittin decreased by four fold in the presence of LTA, the antibacterial effectiveness of [R_4_W_4_] and vancomycin MICs remain unchanged, suggesting that although there is a probable binding of [R_4_W_4_] to LTA, however, the mechanism of action of [R_4_W_4_] may not be contingent on LTA binding affinity, especially at lower concentrations as previously predicted.

## 4. Materials and Methods

### 4.1. Bacterial Strains Utilized

Clinical strains of MRSA and the VRE isolate were taken from a previous collection of isolates. MRSA isolates were genotyped, and their *SCCmec* type was determined. *Escherichia coli* 29522 (*E. coli* 22) was obtained from ATCC (Manassas, VA, USA) and is the standard *E. coli* used in quality control susceptibility testing. The *Bacillus subtilis* (ATCC 21332) and *Clostridioides difficile* (ATCC 9689) strains were acquired from ATCC.

Materials used: *Staphylococcus aureus* (LAC) USA300, *E. coli* 22, Trypticase soy agar (TSA), Cation-adjusted Mueller–Hinton broth (CAMHB) HiMedia Laboratories PVT. Ltd., Mumbai, India; Mueller–Hinton broth, Hardy Diagnostic, (Santa Maria, CA, USA); DISC_3_5 (3,3’-Dipropylthiadicarbocyanine Iodide) fluorescence dye Life Technologies Corporation, (Carlsbad, CA, USA); Vancomycin Hydrochloride, Alfa Aesar, (Ward Hill, MA, USA); Levofloxacin (LVX), TCI America USA.; D+-Glucose Sigma Lifesciences, (St Louis, MO, USA); Melittin Honey bee GenScript Biotech, (Piscataway, NJ, USA); Dimethyl Sulfoxide (DMSO) MP Biomedicals, (Irvine, CA, USA); Nisin Sigma Sciences, (Albuquerque, NM, USA); Absolute Ethanol Fisher bioreagents, (Pittsburgh, PA, USA); sterile inoculating loops, Incubator with shaker, Incubator SheLLab (Cornelius, OR, USA), VMR V-1200 spectrophotometer for culture turbidity measurement at 600 nm, 0.5 McFarland, 15 mL round bottom tubes, 2 mL Eppendorf tubes, Malvern Zetasizer Nano Series Nano-ZS, Malven Zetasizer nanoseries disposable folded capillary cells, 50 mL tube, human serum, Sodium Chloride salt Fisher Scientific (Pittsburgh, PA, USA), Ferric Chloride Fisher Scientific, Calcium Chloride, NBS 96 wells plates, sterile 0.9% HEPES buffer Sigma Aldrich, (St Louis, MO, USA); 0.02 µM pore-size filter, Spectra Max M5 plate reader, Biosafety cabinet Thermo Scientific (West Hills, CA, USA); BSL2, Flow cytometry, Confocal microscope Olympus CX 41, Matrix Assisted Laser Deionization Mass spectroscopy (MALDI) Bruker autoflex speed, Top bench centrifuge Labnet PrismR, Thermos scientific Sorvall Lynx 4000 centrifuge, Reverse-phase HPLC, analytical HPLC, C-18 HPLC column (XBridge BEH130 Prep C18, Waters (Milford, MA, USA); flash chromatography, rotavapor evaporator, software and online tools (Sci-finder, Reaxys, Prism 8, Chemdraw 18.0) Xterra MS C18 column (3.9 × 150 mm, 5 μm, Waters, (Milford, MA, USA USA); Nexera XR, and Prominence Shimadzu LC-20AD XR systems (Shimadzu Corporation, Kyoto, Japan) coupled with Shimadzu LC-MS-2020 mass spectrometer (Shimadzu, Columbia, MD, USA). A two-component LC system consisted of mobile phase A (0.1% formic acid and 10% ACN in water) and mobile phase B (0.1% formic acid and 100% ACN), BODIPY-TR-cadaverine (BC) fluorescent dye, Tris-HCl buffer (50 mM, pH 7.4) G-Biosciences, (St Louis, MO, USA); LTA from *Staphylococcus aureus* and 5 mg Millipore Sigma, (Burlington, MA, USA); VWR pipet tips standard, BODIPY-TR cadaverine 95% Medchem Express, (Monmouth, NJ, USA).

### 4.2. Antibacterial Effectiveness of [R_4_W_4_]

To determine whether the antibacterial activity of [R_4_W_4_] is bacteriostatic or bactericidal against clinical isolates of multidrug-resistant bacterial isolates, MIC and MBC were determined using the microbroth dilution method. Bacteriostatic activity was defined as a ratio of the MBC/MIC greater than four and bactericidal activity as MBC/MIC ratio ≤ 4 [33]. We added aliquots of test samples (256 μg/mL concentration of [R_4_W_4_] or vancomycin as a control prepared in TSB) to the first well of microplates. MHB media was added to the remaining wells. Two-fold serial dilutions were performed, starting with the highest drug concentration in well 1, and the dilution process continued. A bacterial aliquot of 1/150 dilution was prepared with a 0.5 McFarland standard solution from an overnight culture of the tested bacteria isolate. Bacteria were added to the microplate and placed into wells with the drug dilutions. The microplates were incubated overnight for 24 h at 37 °C. [R_4_W_4_] was synthesized and purified, as reported earlier [28].

### 4.3. Evaluation of Time-Kill with [R_4_W_4_] against MRSA 

The MRSA Los Angeles County (LAC) USA300 strain was cultured from −80 °C freezer stock and inoculated on an agar plate overnight. Two bacteria colonies were inoculated in MH broth overnight at 37 °C with shaking at 200 rpm. An aliquot was added to normal saline to achieve a 0.5 McFarland Standard suspension and then diluted 1:150 with MHB. The bacteria sample was plated on an agar plate at time zero. The test samples were prepared by adding [R_4_W_4_] or vancomycin to bacteria to achieve the final sample concentration of (1 × MIC, 2 × MIC, 4 × MIC) using media without antibacterial agents as positive control. 

At the time interval of 0, 4, 8, 12, 18, and 27 h, an aliquot was removed for plating in duplicates. The samples were diluted to minimize excessive CFU counts. The inoculated plates were incubated at 37 °C overnight. The surviving organisms were determined by counting colonies as CFU/plate. The average duplicate counts were multiplied by the dilution factors to arrive at CFU/mL. The microbial reduction was calculated by converting the count to log_10_ scale. The Log CFU/mL was plotted against time for different antibacterial concentrations. 

### 4.4. Salt and Temperature Sensitivity

The effects of cations on the antimicrobial activity of [R_4_W_4_] were determined against MRSA using vancomycin as a control. The bacteria aliquots were treated with MHB media containing the final concentration of sodium chloride (NaCl) (150, 75, 37.5, and 18.75 mM), calcium chloride (CaCl_2_) (8, 4, 2, and 1 mM), and ferric chloride (FeCl_3_) (1, 4, and 8 µM) in 96 well microplates. The MIC of [R_4_W_4_] and vancomycin were determined to determine whether there were changes in the MIC values when treated with different salt concentrations [50]. 

As per temperature sensitivity, the [R_4_W_4_] stock solution (1.15 mg/mL) in a 2 mL Eppendorf microtube was exposed to 100 °C for 10 min. After the thermal exposure, the [R_4_W_4_] antibacterial effectiveness was evaluated against MRSA using a microbroth dilution assay, as explained above.

### 4.5. Synergistic Evaluation of [R_4_W_4_] and Frontline Antibiotics against Bacteria Pathogens by Checkered Plate Assay

An amount of 100 µL of [R_4_W_4_] peptide test solution at a final concentration of 512 µg/mL was added to each well A-G column 1 in plate A using a multi-channel pipette. A 50 µL MH Broth was added to micro-plate well A-G columns 2–9. Using a multi-channel pipette, a two-fold serial dilution was performed by removing 50 µL solution from column 1 and was added to column 2 with thorough mixing [37]. The micro-pipette was discarded after each transfer. The last portion after column 9 was discarded. In another microplate, 100 µL MHB was added to well B-G columns 1–11 using a multi-channel pipette. A 200 µL gentamicin test solution 512 µg/mL was added to row A, columns 1–11. A two-fold serial dilution was performed by removing 100 µL from rows A to B. The last portion was discarded. Using eight multi-channel pipettes, 50 µL of gentamicin test solution was transferred in the ordinate column A-G row 1 to the first microplate. The plate was incubated at 37 °C for 18–24 h [51]. The fractional inhibitory concentration (FIC) of the antibacterial agents and the fractional inhibitory concentration index (FICI) of the two antibacterial agents were determined to evaluate the synergistic interaction [38]. The FICI was interpreted as follows: FICI ≤ 0.5, synergy; 0.5 < FICI ≤ 1, partial synergy; 1 ≤ FICI < 4, additive effect or indifference; 4 ≤ FICI antagonism and calculated from the mean values from multiple experiments. 

### 4.6. Bacterial Resistance to [R_4_W_4_]

MRSA USA300 bacteria from −80 °C freezer stock were cultured on an LB agar plate overnight. One bacteria colony was added to a 6 mL fresh MHB with constant shaking at 200 rpm at 37 °C. The bacteria were transferred daily by inoculating 10 μL of stationary-phase culture into 90 mL of MHB in a 1 mL cap-clipped Eppendorf tube. Then, 20 μL of bacteria aliquot was added to 90 mL of MHB broth, with or without [R_4_W_4_], at a final concentration of the MIC overnight in an autoclave at 37 °C for five transfers. The regrown bacteria were thereafter transferred to broth containing a double concentration of [R_4_W_4_] for about six transfers, or more frequently if the selection strains showed weak growth. The experiment was conducted for 29 serial transfers. Experiments were performed in triplicates. Every time we increased the concentration of [R_4_W_4_], a sample of the induction generation was inoculated into 20% (vol/vol) glycerol and stored at −70 °C. The final MIC values of [R_4_W_4_], vancomycin, and levofloxacin were determined by modified microbroth dilution assay [52].

### 4.7. Evaluation of [R_4_W_4_] Stability to Enzymes in Human Serum and Human Plasma

Fresh human serum (4 mL) was centrifuged at 12,500× *g* for 15 min at 4 °C to remove the lipids. A 25% human serum sample was prepared by adding 375 µL of the human serum supernatant to 1125 µL of RPMI buffer. Then, 129 µL aliquots of 1.28 mg/mL [R_4_W_4_] stock sample were added to 16,371 µL aliquots of 25% human serum to make 16,500 µL aliquot of 25% human serum test sample containing final peptide concentration 100 µg/mL [53,54,55]. The test sample was incubated in a water bath at 37 °C until a predetermined time interval (0, 1, 2, 4, 6, 8.5, 12, and 20 h). At the time intervals, a 150 µL aliquot test sample was added to a 300 µL cocktail solution [ethanol/formic acid (98:2 *v*/*v*) mixture] to cease the reaction. The test samples of aliquots were stored on dry ice for 20 min and then centrifuged at 17,200× *g* for 20 min. For LC-MS analysis, 150 µL supernatant aliquot test samples were stored at −20 °C [54].

For human plasma stability, [R_4_W_4_] was added at a concentration of 1 µM and incubated. At the end of incubation at each of the time points, acetonitrile was added to the incubation mixture, followed by centrifugation. Samples were analyzed by HPLC-MS/MS, and peak areas were recorded for each analyte. The area of precursor compound remaining after each time point relative to the amount remaining at time zero, expressed as percent, is calculated. Subsequently, the half-life (T_1/2_) is estimated from the slope of the initial linear range of the logarithmic curve of the compound remaining (%) versus time, assuming first-order kinetics. The plasma stability experiment was performed at Eurofins Inc. (San Diego, CA, USA) [56].

#### LC-MS Analysis

A 20 μL aliquot of each sample was analyzed using an Xterra MS C18 column (3.9 × 150 mm, 5 μm, Waters, MA, USA), Nexera XR, and Prominence Shimadzu LC-20AD XR systems (Shimadzu Corporation, Kyoto, Japan) coupled with Shimadzu LC-MS-2020 mass spectrometer (Shimadzu, OR, USA). A two-component LC system consisted of mobile phase A (0.1% formic acid and 10% acetonitrile in water) and mobile phase B (0.1% formic acid and 100% acetonitrile). The flow rate was set at 1.0 mL/min. The Xterra MS C18 column at 40 °C was allowed to equilibrate with 95% mobile phase A. Peptides were loaded to the column and washed for 5 min with 95% mobile Phase A. Analytes were separated using a binary gradient of 0–95% mobile Phase B for 25 min. Between 4.0 and 23 min, the mobile phase was set at 55% B. From 25 to 32 min, the mobile phase was set at 95% A. The retention time for [R_4_W_4_] was 12.5 min. MS was conducted in multiple reaction monitoring and positive electrospray ionization mode. Fourteen concentrations of [R_4_W_4_] prepared by two-fold dilution of 200 μg/mL to 0.39 μg/mL were used to prepare calibration curves. The calibration curves were plotted using weighted (1/×2) least-squares linear regression analysis of peptide mass spectra area under the curve (AUC) versus concentration. [R_4_W_4_] was quantified with the most abundant multiple reaction monitoring transitions *m*/*z* 685 [M]^2+^.

### 4.8. Determination of [R_4_W_4_] Surface Charge Neutralization against MRSA Membrane by Zeta Potential Measurements

Stock cultures of the MRSA strain from −80 °C were inoculated on MHB agar plates at 37 °C overnight. Two isolated colonies were inoculated in 6 mL MHB broth and allowed to grow overnight with shaking at 225 rpm. A 100 µL bacteria aliquot was added to a 5 mL fresh MHB, and the suspension was incubated to grow for 2 h at 37 °C to achieve a final bacteria concentration of ~3 × 10^8^ CFU/mL as (A 600 ~0.1). The bacterial suspension was diluted using fresh MHB to 3 × 10^5^ CFU/mL. The bacterial suspension was centrifuged at 11,400× *g* for 10 min and washed twice using 10 mM HEPES buffer, pH 7.4, containing 150 mM NaCl (normal saline).

Test samples of [R_4_W_4_] or vancomycin were prepared at 0.2, 0.4, 0.8, 1.2, and 2.4 × MIC by two-fold serial dilution using HEPES buffer, pH 7.4, containing 150 mM NaCl and then filtered using 0.22 µm pore-size filter. An amount of 100 µLs of test samples was added to 900 µL of the bacterial aliquots using a positive buffer without test samples. 

Meanwhile, the Malvern Zetasizer Nano was set to read samples at 25 °C. The bacterial suspension was dispensed into disposable zeta cells with gold electrodes and allowed to equilibrate for 15 min at 25 °C. The zeta potential value for each sample was calculated from the measured value of electrophoretic mobility using the Smuluchowski equation.

### 4.9. [R_4_W_4_] Membrane Depolarization Assay against MRSA

#### Preparation of Bacteria Cells

Buffer A (5 mM HEPE and 5 mM glucose) and Buffer B (5 mM HEPE, 5 mM glucose, and 0.2 mM EDTA) solutions were prepared and filtered using 0.2 μm filters. Bacteria strains (MRSA and *E. coli*) from a −80 °C freezer were inoculated on an agar plate overnight. Two to three colonies of each bacteria strain were inoculated in 8 mL Cation-Adjusted Muller-Hinton Broth (CAMHB) and allowed to grow overnight. The overnight grown MRSA or *E. coli* bacteria was diluted with a fresh CAMHB (1:100) and incubated at 37 °C for 5 h with shaking at 220 rpm. The resulting bacteria cells at the growth phase were centrifuged at 3700× g at 4 °C for 10 min. The bacteria pellets were washed three times with buffer A and then resuspended in buffer A to an OD_600nm_ of 0.05.

On the other hand, *E. coli* was resuspended in buffer B to an OD_600nm_ of 0.05 to facilitate fluorescence dye DSC_5_ uptake. Then, 20 mL of each bacteria suspension was mixed with 10 μL of 100 μM dye in a dark place for 45 min. After incubation, potassium chloride was added to the two bacteria suspensions to a final concentration. A 2 mL bacterial aliquot was transferred to 1 cm cuvette and antibacterial agents ([R_4_W_4_] or vancomycin or nisin (positive control) and control buffers) to a desired concentration (0.5 × MIC, 1 × MIC, 2 × MIC). The fluorescence intensity was measured at excitation and emission wavelengths of 622 nm and 670 nm, respectively [57].

### 4.10. Lipoteichoic Acid Binding Assay to MRSA LAC USA300

#### 4.10.1. BODIPY-TR-Cadaverine Fluorescent Dye (BC) Displacement Assay

Tris-HCl buffer (6 mL, 50 mM, pH 7.4) mixture containing a final concentration of 50 μg/mL LTA from *Staphylococcus aureus* and 5 μg/mL BC was prepared by adding aliquots of 60 μL and 30 μL of 5 mg/mL LTA from *S. aureus* and 1 g/mL BC stocks to 5.91 mL of Tris-HCl. The mixture was incubated for 4 h in a shaking incubator at 37 °C. Also, the antibacterial agents (melittin, [R_4_W_4_], and vancomycin) were prepared in Tris-HCl buffer (50 mM, pH 7.4) by two-fold serial dilutions to achieve concentration 64, 32, 16, 8, and 2 μg/mL.

An amount of 90 μl aliquots of Tris-HCl buffer (50 mM, pH 7.4) mixture containing a final concentration of 50 μg/mL LTA from *S. aureus* and 5 μg/mL BC were added to 96 microplate wells and 10 μL aliquots of the antibacterial agents were added in triplicate to the mixture. The control was added without antibacterial agents. After adding the antibacterial agents to the mixture, the 96-well microplate was incubated for one hour at 37 °C. The fluorescence intensity was measured at excitation and emission wavelengths 580 nm and 620 nm, respectively, using a Shimadzu Spectro fluorophotometer (Canby, OR, USA) [58].

#### 4.10.2. Bacterial Growth in the Presence of LTA

The interaction of [R_4_W_4_], melittin, and vancomycin with LTA in the presence of MRSA was determined using a microbroth dilution assay. Two-fold serial dilution was used to achieve a 100 μL final concentration of the antibacterial agents in a 96 microplate well. A 360 μL *S. aureus* LTA (Sigma Aldrich stock 5 mg/mL) was added to 17.64 mL MHB containing MRSA (5 x 10^5^ CFU/mL) to achieve the final concentration of *S. aureus* LTA 100 μg/mL. Then, 100 μL aliquots of MHB (MRSA (5 × 10^5^ CFU/mL) and 100 μg/mL *S. aureus* LTA) were added to the 96 microtiter wells containing the antibacterial agents. The microplate was incubated overnight at 37 °C to obtain the MICs. Controls were bacteria grown in the presence of LTA alone and in MHB alone.

## 5. Conclusions

In summary, the outcome of this study provides insight into [R_4_W_4_] as a promising candidate with a therapeutic potential property, including bactericidal activity against clinically resistant bacteria isolates such as Gram-positive MRSA and VRE. [R_4_W_4_] demonstrated stability to human serum degradation and maintained antibacterial activity in physiological salts and at high temperatures. Biophysical assays revealed that [R_4_W_4_] has a remarkable capability of concentration-dependent effect on the MRSA surface charge neutralization (zeta potential) and cytoplasmic membrane depolarization, probably interfering with the biological integrity of the bacteria membrane. Lastly, the mechanism of action of [R_4_W_4_] may be distinct from the glycopeptide vancomycin due to the observed binding affinity for LTA when compared with melittin. Further investigation is required to understand if there is a selective interference of [R_4_W_4_] between bacterial and eukaryotic membranes for optimal therapeutic applications. Therefore, this study collectively contributes to the understanding of [R_4_W_4_] peptide mechanism of action as a promising antimicrobial agent and serves as a strong foundation for further development of optimizing novel antibacterial agents, especially in combating antibiotic resistance.

## Figures and Tables

**Table 1:** [R_4_W_4_] and vancomycin antibacterial activities against bacterial isolates.

Microorganism	Strain	Isolate Source	[R_4_W_4_]			Vancomycin		
			MIC (µg/mL)	MBC (µg/mL)	MBC/MIC	MIC (µg/mL)	MBC (µg/mL)	MBC/MIC
MRSA	Mec 1	Blood	2	4	2	1	2	2
MRSA	Mec II	Blood	4	8	2	2	4	2
MRSA	Mec III	Wound	4	8	2	1	2	2
MRSA	Mec IV	Lung	32	64	2	2	8	4
MRSA	Mec V	Wound	16	64	4	2	4	2
MRSA	Clin 5	Lung	16	32	2	2	2	1
MRSA	Clin 6	Lung	32	32	1	2	8	4
MRSA	LAC	Wound	4	16	4	1	2	2
MRSA (vancomycin hetero-non-susceptible)	MU3	Lung	16	32	2	2	3	1.5
*B. subtilis*	21332	N/A	4	8	2	0.25	0.5	2
*Enterococcus faecium* (VRE)	N/A	Urine	16	64	4	* N/A	N/A	N/A
*C. difficile*	9689	Stool	2	4	2	0.5	1	2

* N/A: not active; Note: MIC and MBC values are the averages of triplicates of three different experiments.

**Table 2:** Lethal rate and microbial log reduction in [R_4_W_4_] and vancomycin against MRSA LAC.

Concentration	[R_4_W_4_]			Vancomycin		
	MIC (µg/mL)	2 × MIC (µg/mL)	4 × MIC (µg/mL)	MIC (µg/mL)	2 × MIC (µg/mL)	4 × MIC (µg/mL)
Lethal rate (LR)	2.48	2.65	2.89	1.02	1.99	2.37
% Reduction	99.67	99.78	99.87	90.50	98.98	99.58

Note: calculating microbial log_10_ reduction: log_10_ reduction (LR) = mean log_10_ (microbial population) − mean log_10_ (surviving population); percent reduction (%) = 100 × (1 − 10^−LR^).

**Table 3:** Fractional inhibitory concentration index of [R_4_W_4_] and gentamicin.

	[R_4_W_4_]		Gentamicin					Integrative Category
Microorganism	MIC_A_ (µg/mL)	MIC_(A+B)_ (µg/mL)	MIC_B_ (µg/mL)	MIC_(B+A)_ (µg/mL)	FIC_A_	FIC_B_	FICI	
MRSA USA300	6	1.5	0.09	0.05	0.25	0.56	0.81	Partial synergy
*E. coli* 25922	32	1.5	0.75	0.19	0.05	0.25	0.30	Synergy

Where A = [R_4_W_4_], B = gentamicin, MIC = minimum inhibitory concentration, FIC = fractional inhibitory concentration, FICI = fractional inhibitory concentration index (FICI) was interpreted as follows: FICI ≤ 0.5, synergy; 0.5 < FICI ≤ 1, partial synergy; 1 ≤ FICI < 4, additive effect or indifference; 4 ≤ FICI, antagonism [31].

## Data Availability

The data presented in this study are available in the article.
